# Supplementary material for: Outflow of cerebrospinal fluid is predominantly through lymphatic vessels and is reduced in aged mice
Source: Nat Commun. 2017 Nov 10;8:1434. doi: 10.1038/s41467-017-01484-6 (PMC5681558; doi:10.1038/s41467-017-01484-6)
Supplement: Supplementary file 1 — Supplementary Information [file 41467_2017_1484_MOESM1_ESM.pdf]

Supplementary Figures and Legends

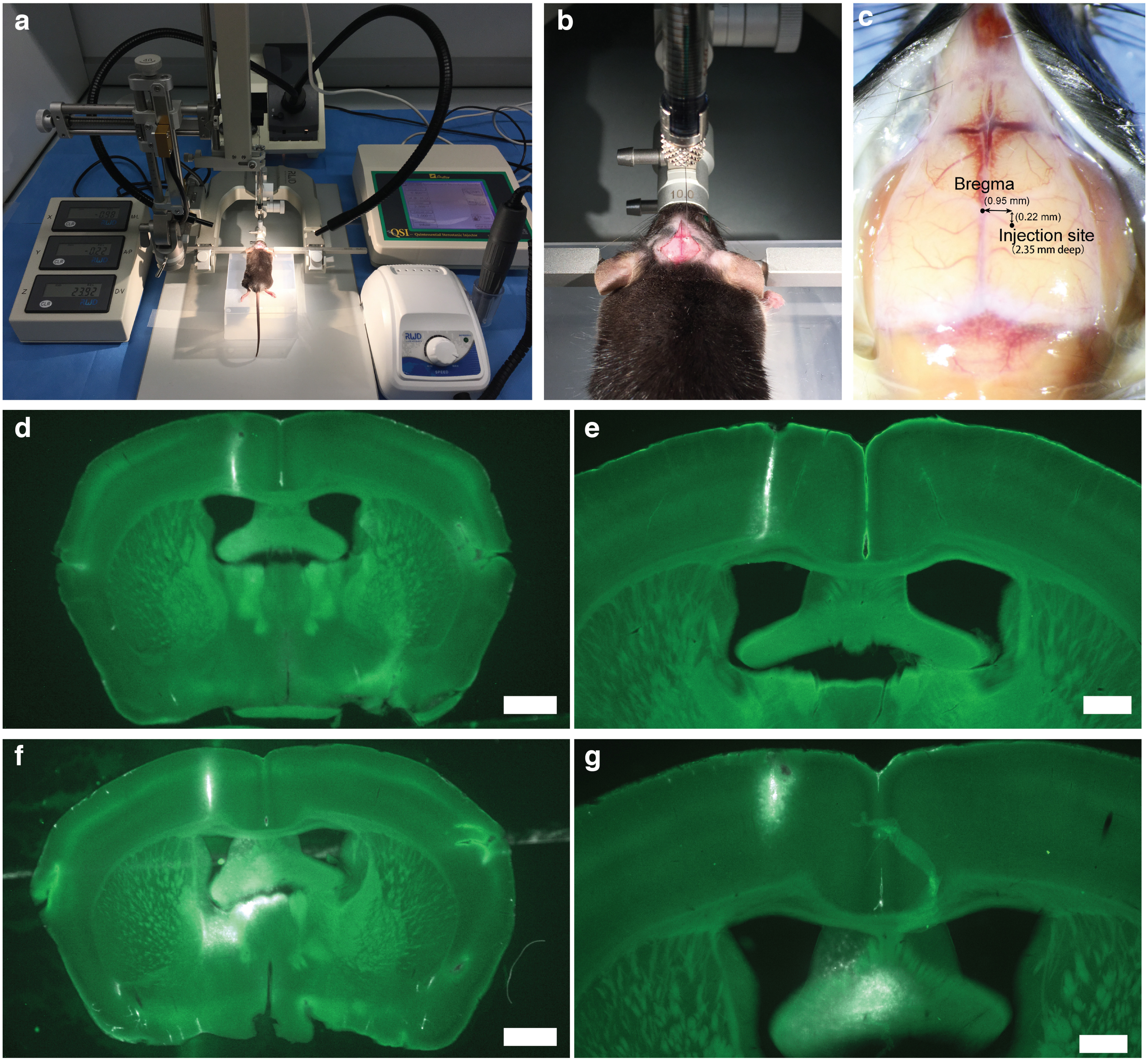

Supplementary Figure 1. Setup and coordinates for stereotactic infusions and quality check for needle placement. (a) The stereotactic setup for infusion into right lateral ventricle including syringe pump, stereotactic frame and dental drill. **(b)** Detailed image showing the needle penetration site. **(c)** The coordinates for the needle penetration as measured from the bregma. **(d-e)** Brain sections of successful infusions. Scale bar in (d): 1 mm, in (e): 500 µm. **(f-g)** Brain sections of poor infusions showing tracer remaining within brain parenchyma. Scale bar in (f): 1 mm, in (g): 500 µm.

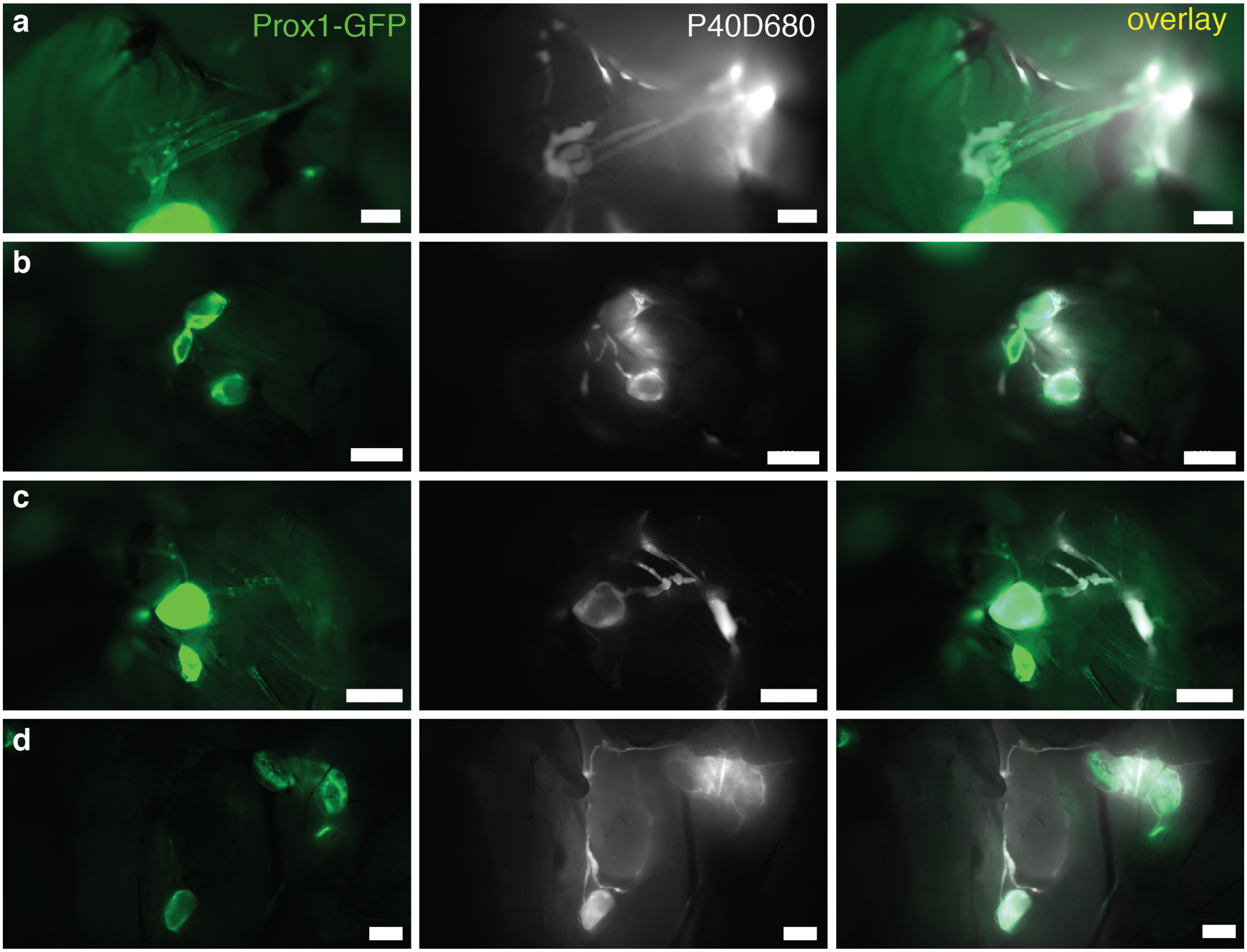

Supplementary Figure 2. Complexity of lymphatic routes near the deep cervical lymph nodes. (a) Representative image of a Prox1-GFP mouse with many deep cervical afferent lymphatic vessels originating from the region of the tympanic bulla. Scale bars: 500 µm. **(b)** Representative image of a Prox1-GFP mouse with three deep cervical lymph nodes on the left side. Scale bars: 1 mm. **(c)** Representative image of a Prox1-GFP mouse with a collecting lymphatic vessel that skips the deep cervical lymph node. Scale bars: 1 mm. **(d)** Representative image of a Prox1-GFP mouse with a lymphatic connection from the deep cervical region to the mandibular lymph nodes. Scale bars: 1 mm.

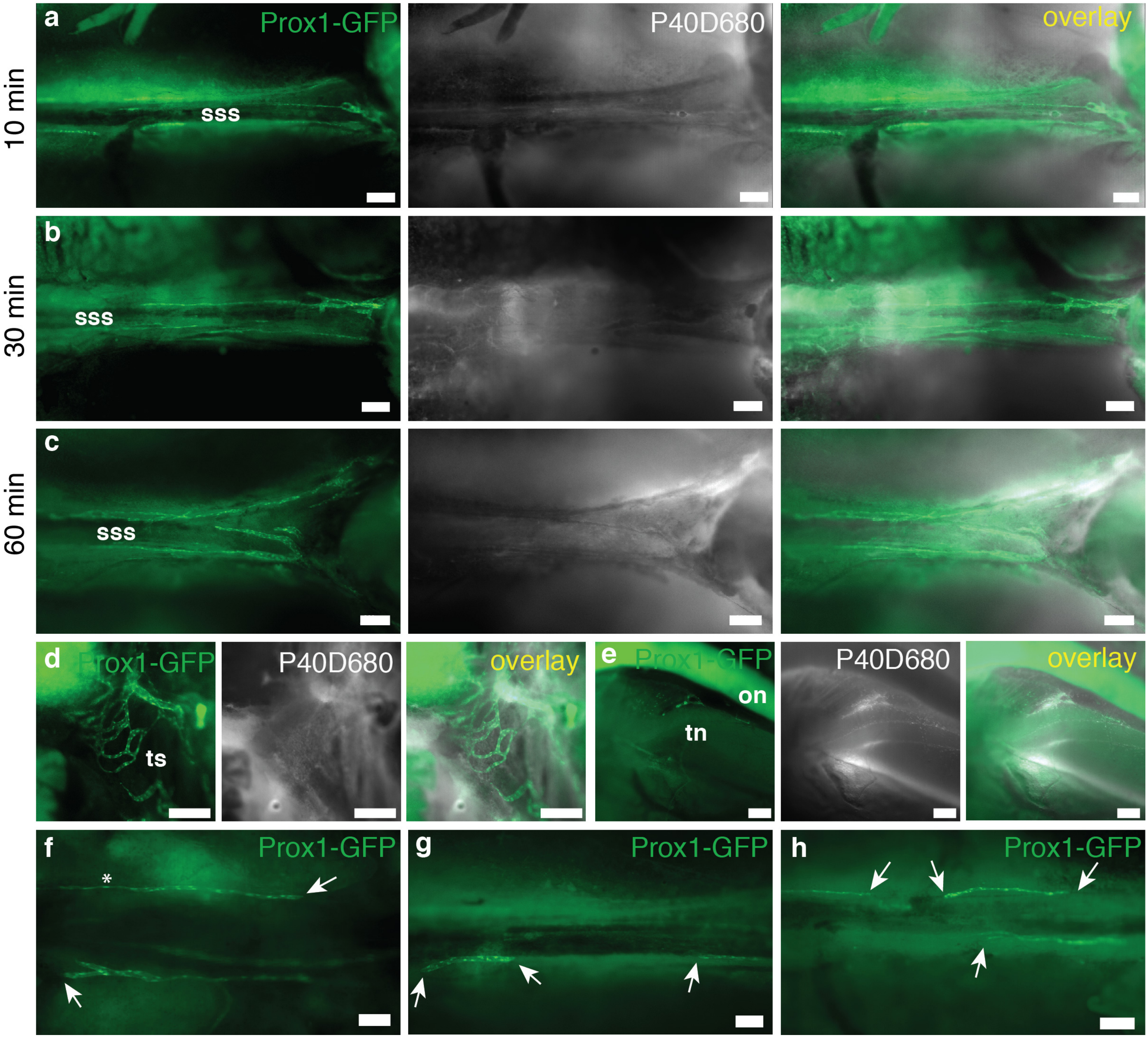

Supplementary Figure 3. Lack of uptake of P40D680 tracer into dura mater lymphatic vessels of the meninges. Representative images of Prox1-GFP mice with valveless lymphatic vessels in the dura mater near the superior sagittal sinus (sss) with no obvious P40D680 uptake 10 min **(a)**, 30 min **(b)** and 60 min **(c)** after intraventricular infusion. Scale bars: 200 μm. Number of mice examined: n=5 (10 min), n=7 (30 min), n=7 (60 min). **(d)** Representative image of a Prox1-GFP mouse with a dense network of valveless lymphatic vessels in the dura mater near the transverse sinus (ts) but no apparent P40D680 uptake. Scale bars: 200 μm. **(e)** Representative image of a Prox1-GFP mouse with a dura mater lymphatic vessel close to the optic nerve. P40D680 tracer is in close proximity to the vessel but does not appear to be within it. Perineural flow appears around the trigeminal (tn) and optic nerves (on). Scale bars: 500 μm. **(f-h)** Representative images of discontinuous dural lymphatic vessels. Arrowheads indicate blind ends of dural lymphatic vessels. Star indicates a cord of individual lymphatic endothelial cells rather than a vessel-like structure. Scale bar in f and g: 100 μm. Scale bar in h: 200 μm.

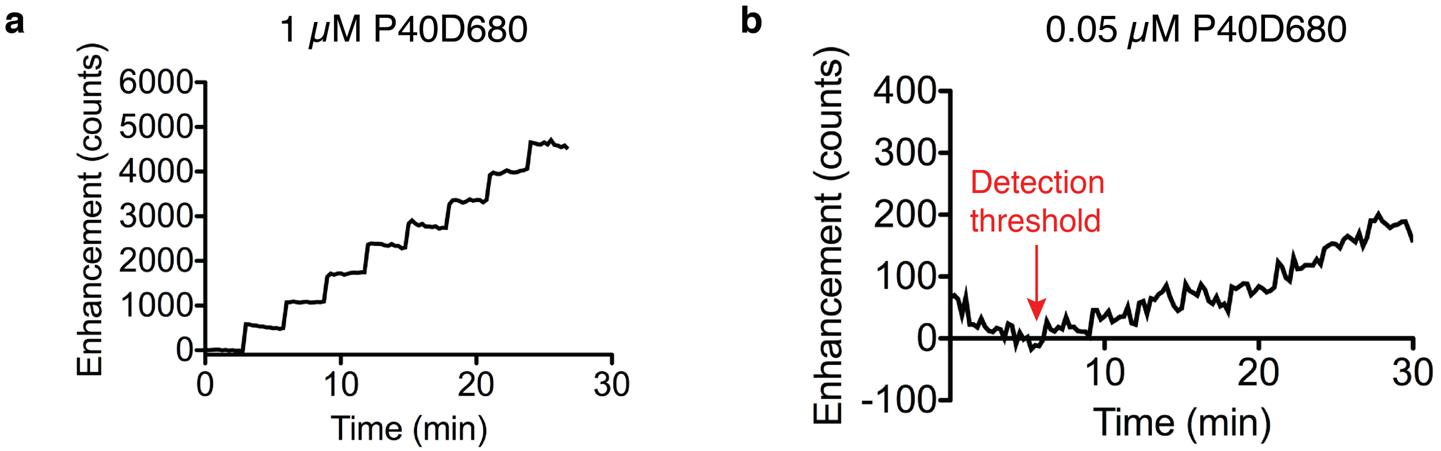

Supplementary Figure 4. Determination of tracer sensitivity of detection in blood and dose calibration of P40D680. Tail vein catheters were implanted into albino C57BL/6J-*Tyr*$^{c\text{-}J}$ mice to measure the saphenous vein signal response to known bolus intravenous infusions of tracer. **(a)** Plot showing saphenous vein signal enhancement during stepwise infusions of 10 µL of 1 µM P40D680 every three min. Each bolus represents 2% of the dose that is infused into the lateral ventricle. Note linear relationship of P40D680 dose with the signal enhancement in blood. Representative of n=3 mice. **(b)** Plot showing saphenous vein signal during stepwise infusions of 10 µL of 0.05 µM P40D680 every three min. Detection threshold indicates time point where signal increase is first detected after two infusions of 0.05 µM P40D680. With a ventricular infusion of 2.5 µL of 200 µM P40D680, the detection threshold in systemic blood would represent 0.2% of the dose. Representative of n=3 mice.

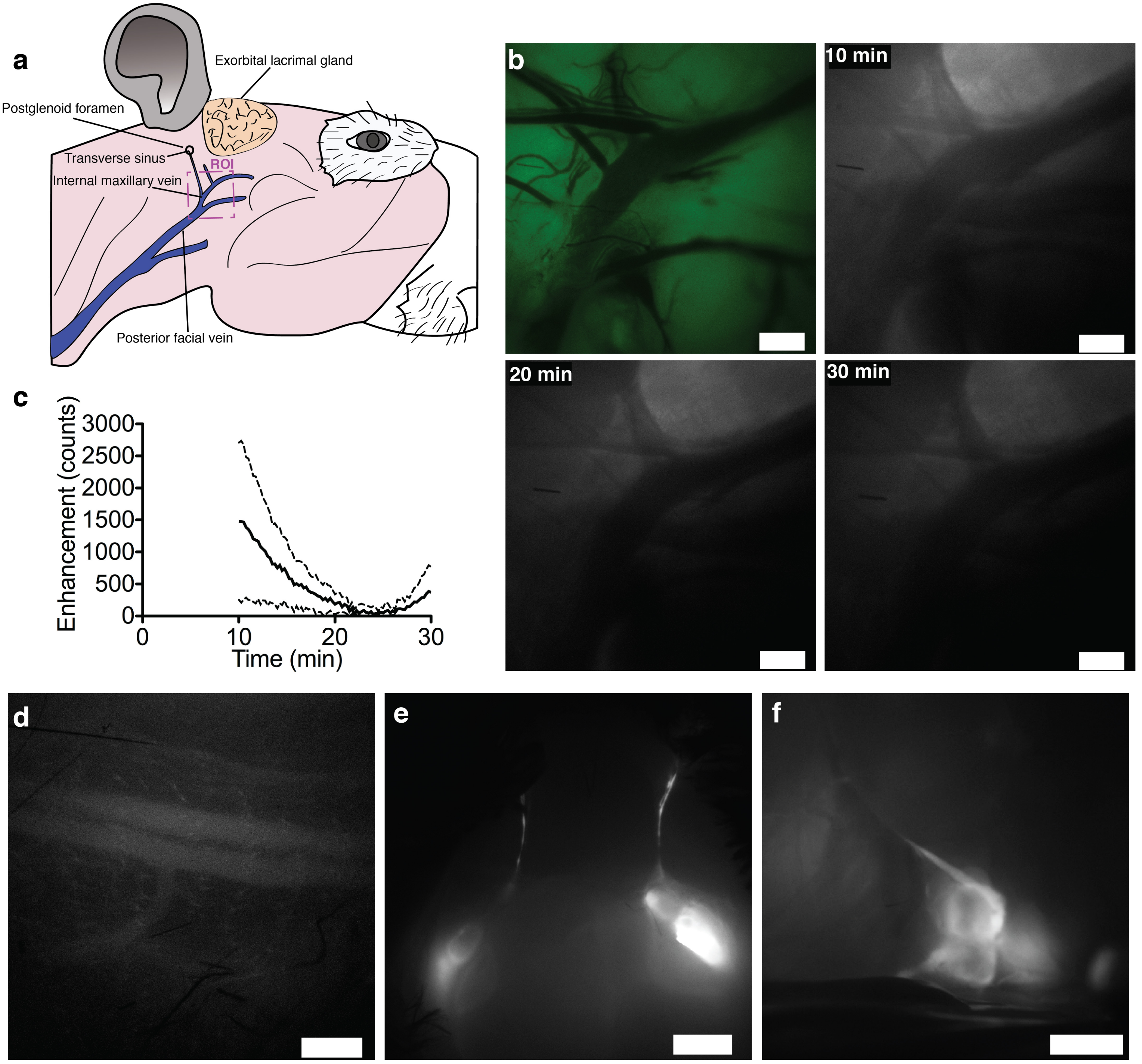

Supplementary Figure 5. Monitoring of signal of the posterior facial vein after intraventricle infusion. (a) Scheme showing region of interest (ROI) for the venous signal monitoring. **(b)** Representative autofluorescence picture on GFP channel showing the blood vessel network and video frames showing the absence of signal from P40D680 tracer within the posterior facial vein at 10 min, 20 min and 30 min after infusion. Scale bar: 500 µm. Note P40D680 signal that is apparent through the skull that declines over time. **(c)** Plot from n=5 mice demonstrating the loss of intracranial P40D680 signal over time and a slight increase in signal beginning at around t = 25 min after infusion. **(d)** Representative image showing the detected signal from P40D680 tracer from saphenous vein at 30 min after infusion. Scale bar: 500 µm. **(e)** Representative image showing the detected signal from P40D680 tracer from submandibular lymph nodes at 30 min after infusion. Scale bar: 2 mm. **(f)** Representative image showing the detected signal from P40D680 tracer from deep cervical lymph nodes at 30 min after infusion. Scale bar: 1.5 mm.

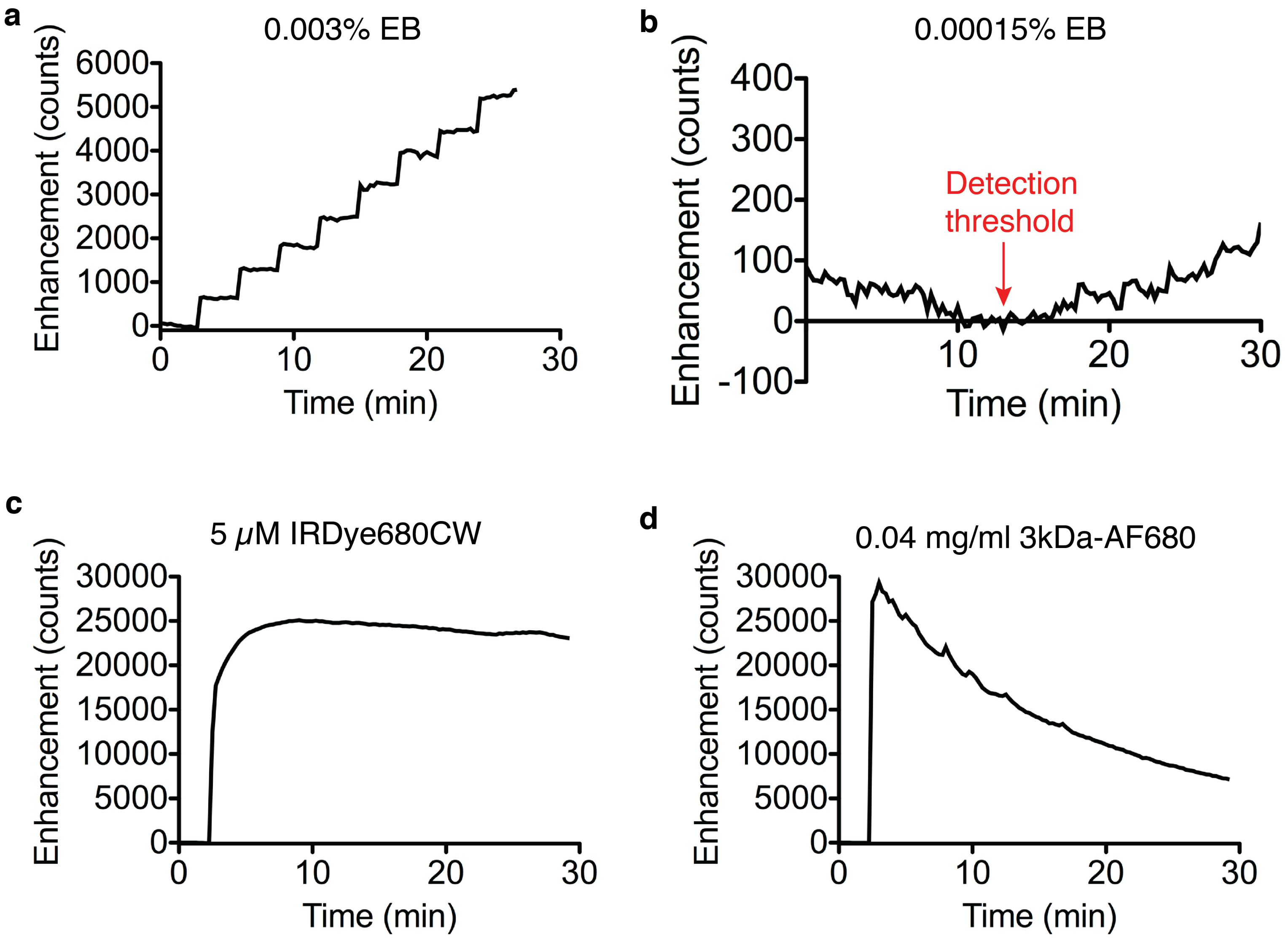

Supplementary Figure 6. Demonstration of tracer sensitivity of detection at saphenous region of different small molecular tracers and dose calibration of EB. Tail vein catheters were implanted into albino C57BL/6J-*Tyr*$^{c\text{-}J}$ mice to measure saphenous vein signal response to known intravenous bolus infusions of tracer. **(a)** Plot showing saphenous vein signal enhancement during stepwise infusions of 10 µL of 0.003% EB. Each bolus represents 2% of the dose that is infused into the lateral ventricle. Note linear relationship of EB dose with the signal enhancement in blood at similar enhancement values as P40D680 (Supplementary Figure 4a). **(b)** Plot showing saphenous vein signal during stepwise infusions of 10 µL of 0.00015% EB. Detection threshold indicates time point where signal increase is first detected after four infusions. With a ventricular infusion of 2.5 µL of 0.6% EB, the detection threshold in systemic blood would represent 0.4% of the dose. **(c)** Plot showing signal enhancement at saphenous region during tail vein injection of 100 µL of 5 µM IRDye680CW at t = 3 min. Tracer rapidly leaked from blood vessels in the skin and was retained over time. **(d)** Plot showing signal enhancement at saphenous region during tail vein injection of 100 µL of 0.04 mg/ml 3kDa-AF680 at t = 3 min. Tracer rapidly leaked from blood vessels in the skin but note decrease in signal over time due to photobleaching effects. Each plot is representative of n=3 experiments.

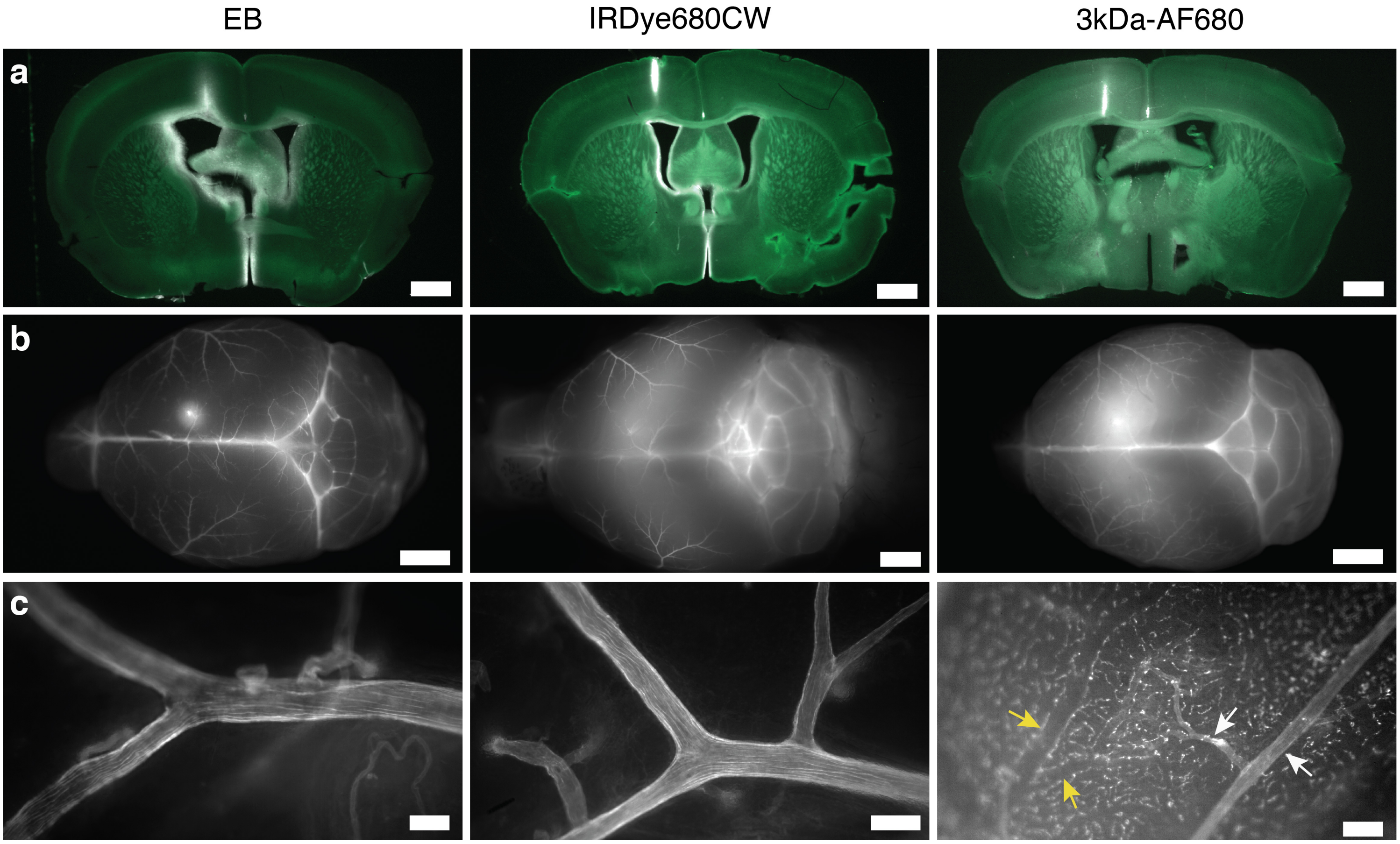

Supplementary Figure 7. Retention of different small molecular tracers within the CNS after intraventricular infusion. (a) Representative picture of cross section of the brain showing diffusion of EB into the parenchyma from the lateral and third ventricles. Scale bars: 1 mm. Less diffusion was seen with IRDye680CW and diffusion was not observed with 3kDa-AF680. Images were acquired at t = 60 min after lateral infusion. **(b)** Representative images of the brain dorsal surface at 60 min after lateral ventricle infusion of EB, IRDye680CW and 3kDa-AF680. Scale bars: 2 mm. **(c)** Detailed representative image of a brain section showing binding of EB and IRDye680CW to arteries at the circle of Willis and the spread of 3kDa-AF680 into pia mater tissue between arteries (white arrows) and veins (yellow arrows). Scale bars: 100 µm. All images representative of n=4 mice per tracer.

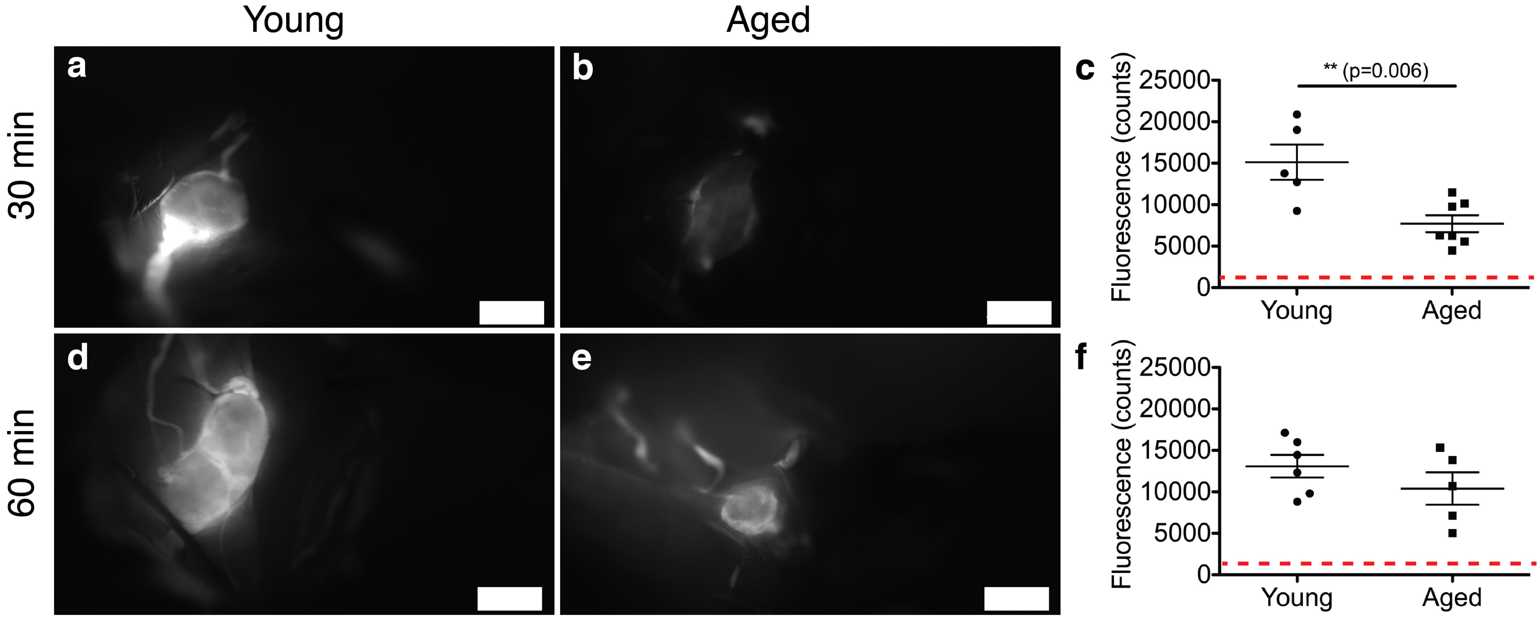

Supplementary Figure 8. Tracer flow in young and aged mice to draining lymph nodes in the neck. (a-b) Representative pictures of tracer within the left deep cervical lymph nodes at 30 min after P40D680 infusion into the right lateral ventricle in young (n=5) and aged (n=7) mice. Scale bars: 1 mm. **(c)** Quantification of fluorescent signal of deep cervical lymph nodes at t = 30 min after infusion. Red dashed line indicates baseline levels in uninjected mice. Data are the mean ± SD. ** $p<0.01$ (2-tailed Student's t-test). **(d-e)** Representative pictures of tracer within the left deep cervical lymph nodes at 60 min after P40D680 infusion into the right lateral ventricle in young (n=6) and aged (n=5) mice. Scale bars: 1 mm. **(f)** Quantification of fluorescent signal of deep cervical lymph nodes at t = 60 min after infusion. Red dashed line indicates baseline levels in uninjected mice. Data are the mean ± SD.

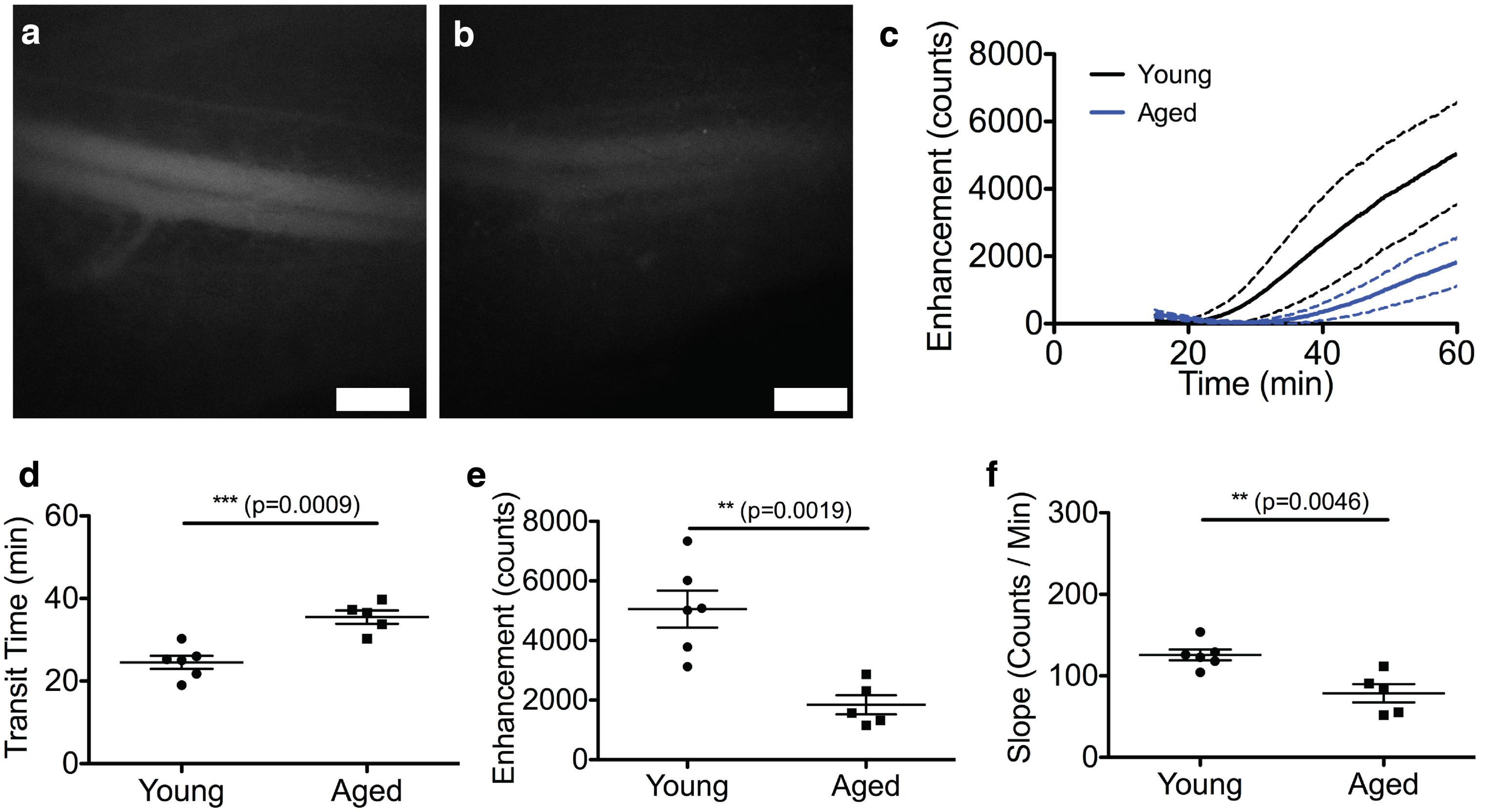

Supplementary Figure 9. Dynamics of CSF transport to blood of P40D680 tracer in young and aged mice after infusion into cisterna magna. (a) Representative image of the saphenous vein in a young (2-month-old) mouse 60 min after cisterna magna infusion of P40D680. **(b)** Representative image of the saphenous vein in an aged (18-month-old) mouse at the same time point. Scale bars: 500 µm. **(c)** Saphenous vein signal enhancement plots of young (n=6) and aged mice (n=5). Solid line indicates mean, dashed lines indicate SD. **(d)** Quantification of transit time. **(e)** Quantification of signal enhancement at 60 min. **(f)** Quantification of slopes of the signal enhancement from 45 to 60 min. ** $p<0.01$, *** $p<0.001$ (2-tailed Student's t-test). Data are mean ± SD.

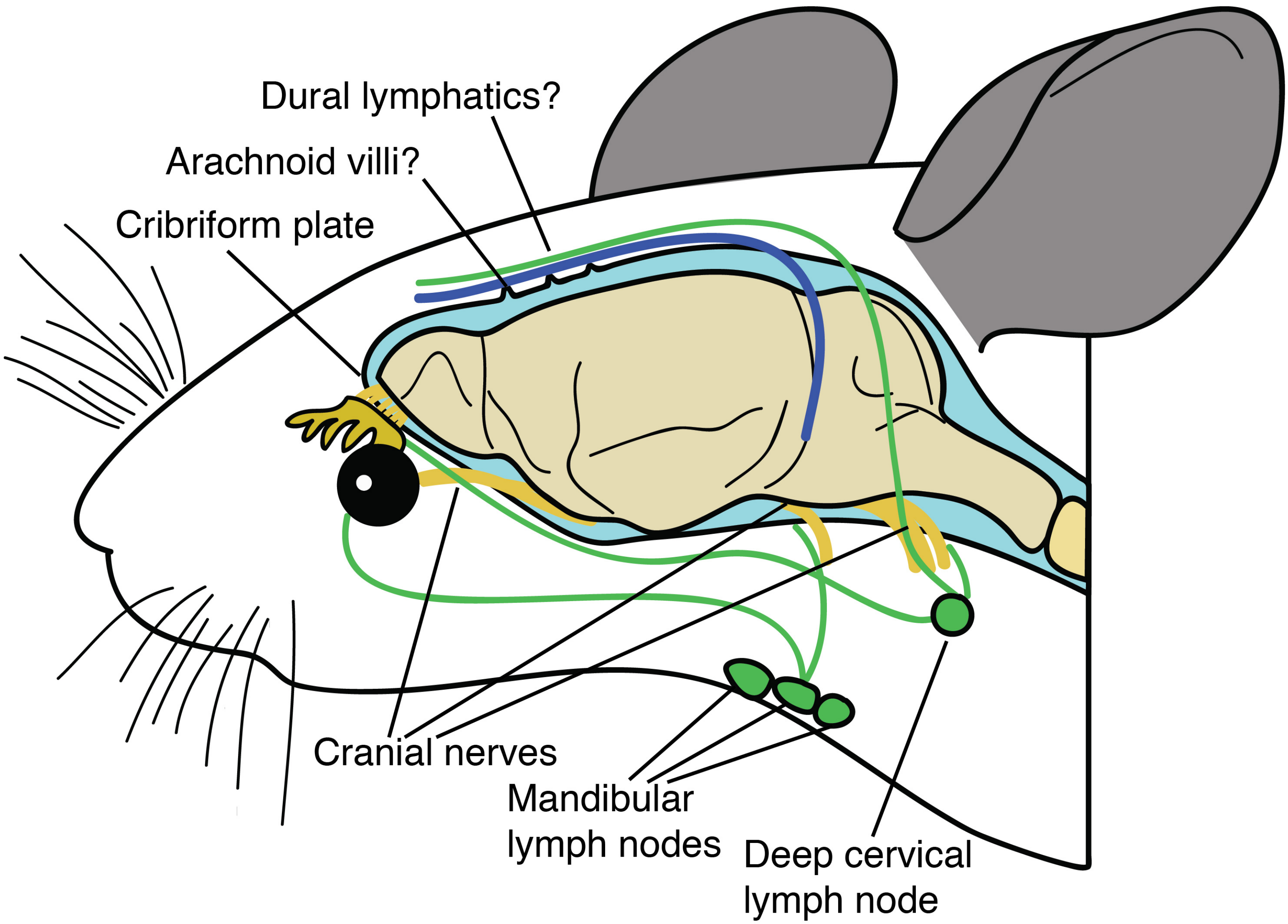

Supplementary Figure 10. Overview scheme of CSF outflow. From the subarachnoid spaces (light blue), CSF outflow occurs at several perineural exits of the skull to reach lymphatic vessels that drain to mandibular and deep cervical lymph nodes of the neck. Spinal lymphatic outflow may also occur. Cranial nerves are shown in yellow and lymphatic vessels are shown in green. The contributions of the arachnoid villi and the dural lymphatic vessels still remain to be elucidated.
