# Supplementary material for: Outflow of cerebrospinal fluid is predominantly through lymphatic vessels and is reduced in aged mice
Source: Nat Commun. 2017 Nov 10;8:1434. doi: 10.1038/s41467-017-01484-6 (PMC5681558; doi:10.1038/s41467-017-01484-6)
Supplement: Supplementary file 3 — Description of Additional Supplementary Files [file 41467_2017_1484_MOESM3_ESM.pdf]

Description of Additional Supplementary Files

File Name: Supplementary Movie 1
Description: **Saphenous vessel imaging after intraventricular infusion of P40D680.** Noninvasive imaging of the saphenous vein region on the left leg of a Prox1-GFP mouse starting 5 min after intraventricular infusion of 2.5 µL of 200 µM P40D680. No P40D680 signal is seen at start of movie at t = 0 min, signal becomes first apparent by eye at around t = 25 min and continues to increase throughout the experiment. Images are acquired at 1 frame per 15 s at 25x magnification.

File Name: Supplementary Movie 2
Description: **Mandibular lymph node and afferent collecting lymphatic vessel imaging after intraventricular infusion of P40D680.** Noninvasive imaging of the neck of a Prox1- GFP mouse starting 5 min after intraventricular infusion of 2.5 µL of 200 µM P40D680. No P40D680 signal is seen at start of movie at t = 0 min, signal becomes first apparent in lymphatic vessels and lymph nodes at around t = 7 min (12 min after infusion). Note signal in lymph node on right side of movie (left side of mouse) is apparent before superficial afferent lymphatic vessel is observed, indicating a connection from the deep cervical region. Images are acquired at 1 frame per 1 s at 6x magnification.

File Name: Supplementary Movie 3
Description: **Posterior facial vein imaging after intraventricular infusion of P40D680.** Imaging of ROI shown in Supplementary Figure 5a of a C57BL/6J-*Tyr*$^{c\text{-}J}$ mouse starting 10 min after intraventricular infusion of 2.5 µL of 200 µM P40D680. Background signals decline over time from intracranial P40D680, however, no signal is apparent within the posterior facial vein.

File Name: Supplementary Movie 4
Description: **Saphenous vessel signals in a young (2-month-old) mouse after intraventricular infusion of P40D680.** Imaging of a region on the left leg of a young mouse starting 5 min after intraventricular infusion of 2.5 µL of 200 µM P40D680. No P40D680 signal is seen at start of movie at t = 0 min, signal becomes first apparent at around t = 22 min and continues to increase throughout the experiment. Images are acquired at 1 frame per 15 s at 25x magnification.

File Name: Supplementary Movie 5
Description: **Saphenous vessel signals in an aged (18-month-old) mouse after intraventricular infusion of P40D680.** Imaging of a region on the left leg of an aged mouse starting 5 min after intraventricular infusion of 2.5 µL of 200 µM P40D680. No P40D680 signal is seen at start of movie at t = 0 min, signal becomes first apparent at around t = 32 min and enhances to a lesser degree than in young mice over time. Images are acquired at 1 frame per 15 s at 25x magnification.
